# Effects of Health Qigong on quality of life, physical function, and mental health in older adults: a systematic review and meta-analysis of randomized controlled trials

**DOI:** 10.3389/fpubh.2026.1916466

**Published:** 2026-08-26

**Authors:** Shi-han Liang, Ming-yuan Zhao, Si-min Huang, Qing-Fa Dou, Ya-kai Zhang

**Affiliations:** 1 Graduate School, Harbin Sport University, Harbin, Heilongjiang, China; 2 Faculty of Physical Education, Harbin Sport University, Harbin, Heilongjiang, China; 3 College of Physical Education and Health, Zhaoqing University, Zhaoqing, Guangdong, China

**Keywords:** Health Qigong, older adults, physical function, mental health, quality of life, systematic review, meta-analysis

## Abstract

**Background:**

Health Qigong is widely used for health promotion in older adults. This study aimed to systematically evaluate its effects on physical function, psychological well-being, and quality of life (QoL), alongside the moderating effects of intervention characteristics.

**Methods:**

Seven databases were systematically searched (up to May 2025). Twenty-two randomized controlled trials involving 1,791 older adults were included. Risk of bias was assessed using Cochrane RoB, and meta-analyses were performed using RevMan 5.4 and Stata 18.0.

**Results:**

Health Qigong significantly improved 6-min walk distance (MD = 18.05 m, *p* = 0.03), balance (SMD = 1.70, *p* = 0.004), depressive symptoms (SMD = −0.64, *p* = 0.004), and QoL (SMD = 0.76, *p* = 0.03), but showed no significant effect on activities of daily living (ADL). Subgroup analyses suggested that while Qigong’s benefits on most outcomes were comparable to conventional exercise, it may have greater potential in enhancing QoL. Additionally, regimens with high frequency (≥3 sessions/week), long duration (≥12 weeks), and moderate session length (<60 min) appeared to maximize benefits for alleviating depression and improving QoL.

**Conclusion:**

Health Qigong is an effective intervention for promoting multidimensional health in older adults. While most of its benefits are comparable to conventional exercise, Health Qigong may offer unique advantages in improving QoL. For clinical and community practice, a regular regimen of “high frequency, long duration, and moderate session length” is recommended, with flexible adaptations based on individual health conditions.

**Systematic review registration:**

https://www.crd.york.ac.uk/PROSPERO/view/CRD420251112609.

## 1 Introduction

The accelerating pace of global population aging is one of the most critical social trends of the 21st century (1). With increased life expectancy, the focus of geriatric health has shifted from disease control to a multidimensional perspective encompassing overall well-being, physical function, and mental health. quality of life (QoL) has gained increasing prominence as a key outcome reflecting an individual’ s subjective experience. The World Health Organization defines QoL as an individual’ s perception of their position in life within their cultural and value systems, and in relation to their goals, expectations, standards, and concerns (2). For older adults, QoL is closely intertwined with physical function, psychological state, and social participation. Therefore, identifying safe and scalable interventions to improve these dimensions is crucial for promoting healthy aging (3, 4).

Health Qigong is rooted in traditional Chinese wellness culture and integrates slow movements with breath regulation and focused attention (5). Characterized by low-to-moderate intensity, gentle motions, and ease of learning, it is highly suitable for community-based implementation and can be tailored to individual functional levels, making it particularly applicable to older adults with limited exercise tolerance (6–8).

Although existing studies suggest potential health benefits of Health Qigong for older adults (9), current evidence from systematic reviews and meta-analyses remains markedly limited and insufficient to fully inform community-based practice. First, previous reviews have frequently combined Health Qigong with Tai Chi, yoga, or other mind–body exercises, thus limiting the identification of the specific effects of Health Qigong (10, 11). Second, most studies have concentrated on specific patient groups or frail subgroups, focusing on physical indicators such as balance and gait. Consequently, multidimensional evidence assessing QoL, physical function, and mental health across a broad older population—including both healthy individuals and those with underlying conditions—remains scarce (12, 13). Third, although a few recent meta-analyses have begun to differentiate between control types or explore the moderating effects of intervention parameters (14), these efforts have largely targeted specific diseases and have yet to systematically examine implementation characteristics such as frequency, session duration, and total program length in the broader older population.

Therefore, this systematic review and meta-analysis aims to comprehensively evaluate the effects of Health Qigong on QoL, physical function, and mental health in older adults, encompassing a broad population with varying baseline health statuses rather than being limited to specific diseases. We will distinguish between active and non-active control groups and conduct exploratory analyses of intervention frequency, session duration, and total program length for QoL and mental health outcomes. This study seeks to assess the potential role of Health Qigong in promoting functional health in older adults, thereby informing the dissemination of exercise interventions and community-based public health practice within the framework of active aging.

## 2 Research design and methods

### 2.1 Protocol and registration

This systematic review was conducted in strict accordance with the Preferred Reporting Items for Systematic Reviews and Meta-Analyses (PRISMA) guidelines (15). Furthermore, the study protocol was registered with the International Prospective Register of Systematic Reviews (PROSPERO) under the registration number CRD420251112609.

### 2.2 Inclusion and exclusion criteria

Studies were included if they met the following criteria: (1) participants: older adults aged ≥60 years (according to the World Health Organization definition); (2) intervention: Health Qigong as the sole additional structured intervention; (3) comparison: control groups receiving usual care, cognitive training, health education, other non-exercise activities, or exercise interventions of a different type or intensity; (4) outcomes: reporting at least one outcome measure related to physical function (e.g., 6-min walk test, timed up and go test), mental health (e.g., depression scale scores, subjective well-being scale), or quality of life (e.g., global quality of life scale, sleep quality scale, pain score); (5) study type: randomized controlled trial (RCT); (6) language: published in Chinese or English.

Studies were excluded if they met any of the following criteria: (1) participants included individuals with severe functional impairment (e.g., very low scores on activities of daily living scales or a clinical diagnosis of disability), significant cognitive decline (e.g., Mini-Mental State Examination scores below education-adjusted norms), a history of limb amputation, or recent major acute illness (e.g., acute-phase myocardial infarction or stroke); (2) The intervention was a mixed program combining Health Qigong with other exercises; (3) The full text or credible data were unavailable; (4) The publication was a duplicate, conference abstract, case report, dissertation, commentary, review, systematic review, study protocol, or content unrelated to the current review.

### 2.3 Search strategy

We systematically searched seven electronic databases (Web of Science, PubMed, Embase, The Cochrane Library, CNKI, Wanfang Data, and VIP from their inception to May 2025). The search strategy in PubMed combined MeSH terms and keywords as follows: (1) “Qigong” OR “Qi Gong” OR “Ch’i Kung”; (2) “Aged” OR “Elderly” OR “Aging”; (3) “Health” OR “Health Status” OR “Physical Health” OR “Mental Health” OR “Quality of Life”; (4) “Randomized Controlled Trial” OR “RCT” OR “Randomized.”

The search structure combined these sets with the Boolean operator AND: (1) AND (2) AND (3) AND (4). For the Chinese-language databases, we used equivalent Chinese keywords. Notably, synonyms for “Qigong” were not used in these Chinese database searches.

### 2.4 Study selection and data extraction

Two reviewers (S. H. L. and Q. H. D.) independently performed the study selection and data extraction. Initially, they independently screened titles and abstracts to identify potentially eligible studies. Full texts of these studies were then retrieved and independently assessed for final inclusion. Any disagreements during either stage were resolved through discussion; if consensus could not be reached, a third reviewer (M. Y. Z.) was consulted for arbitration. Data were extracted using a standardized, pre-designed form. The extracted information included: first author, publication year, country, sample size, participant characteristics, intervention details (specific Qigong form, frequency, duration, and program length), comparison conditions, outcome measures and assessment time points, and main findings.

### 2.5 Quality assessment

The methodological quality of the included studies was evaluated using the Cochrane Risk of Bias tool (RoB), in accordance with the recommendations in the Cochrane Handbook (16). This assessment was independently conducted by two reviewers (S. H. L. and Q. H. D.). Any disagreements were resolved through discussion or, if necessary, by consultation with a third reviewer (M. Y. Z.). Given the nature of the exercise intervention and the requirement to fully inform participants about precautions prior to implementation, it was not possible to blind participants and the personnel delivering the intervention across all included studies. Consequently, this specific domain was excluded from the evaluation. The primary assessment therefore focused on the following domains: random sequence generation, allocation concealment, blinding of outcome assessment, completeness of outcome data, selective reporting of results, and other potential biases.

Following the assessment, an overall risk of bias judgment was assigned to each study. A study was judged to have a “low risk of bias” overall if it was rated as low risk in all domains. It was judged to have an “unclear risk of bias” if it was rated as unclear risk in at least one domain but was not at high risk in any domain. A study was judged to have a “high risk of bias” overall if it was rated at high risk in at least one domain. Review Manager (Version 5.4) was used to generate the risk of bias summary figures based on the RoB assessments.

### 2.6 Data analysis and synthesis

Statistical analyses for the meta-analysis were performed using Review Manager (Version 5.4). For continuous outcomes, the effect size was calculated as the mean difference (MD) with a 95% confidence interval (CI). If different units of measurement were used across studies for the same outcome, the standardized mean difference (SMD) was employed as the effect measure (16). Statistical heterogeneity among studies was assessed using the *I*^2^ statistic, with predefined thresholds for interpretation: *I*^2^ < 50% indicating low heterogeneity, 50% ≤ *I*^2^ ≤ 75% indicating moderate heterogeneity, and *I*^2^ > 75% indicating high heterogeneity (17). The choice of analytic model was based on the heterogeneity results. A random-effects model was used if substantial heterogeneity was detected (*I*^2^ ≥ 50%); otherwise, a fixed-effects model was applied. For each outcome, a meta-analysis was conducted if at least two studies were included. If a subgroup contained only one study, its effect size was presented in the forest plot but was reported descriptively in the text without statistical pooling or inference. To investigate potential differences in intervention effects based on the comparison context, a pre-specified subgroup analysis was performed according to the type of control group (exercise control vs. non-exercise control). For multi-arm trials, the Health Qigong group was compared separately with each control group and assigned to different subgroups by control type, without splitting the Health Qigong sample size. Sensitivity analyses were conducted to evaluate the robustness of the findings. This involved sequentially excluding studies with low methodological quality and using a leave-one-out method, applying a fixed-effects model to assess the influence of individual studies. Publication bias was assessed using Stata (Version 18.0). For each meta-analysis, Egger’s test was performed to quantitatively evaluate potential publication bias. The significance level for all statistical tests was set at *α* = 0.05 (i.e., *p* < 0.05 was considered statistically significant) (18).

## 3 Results

### 3.1 Search results

**Figure 1:**
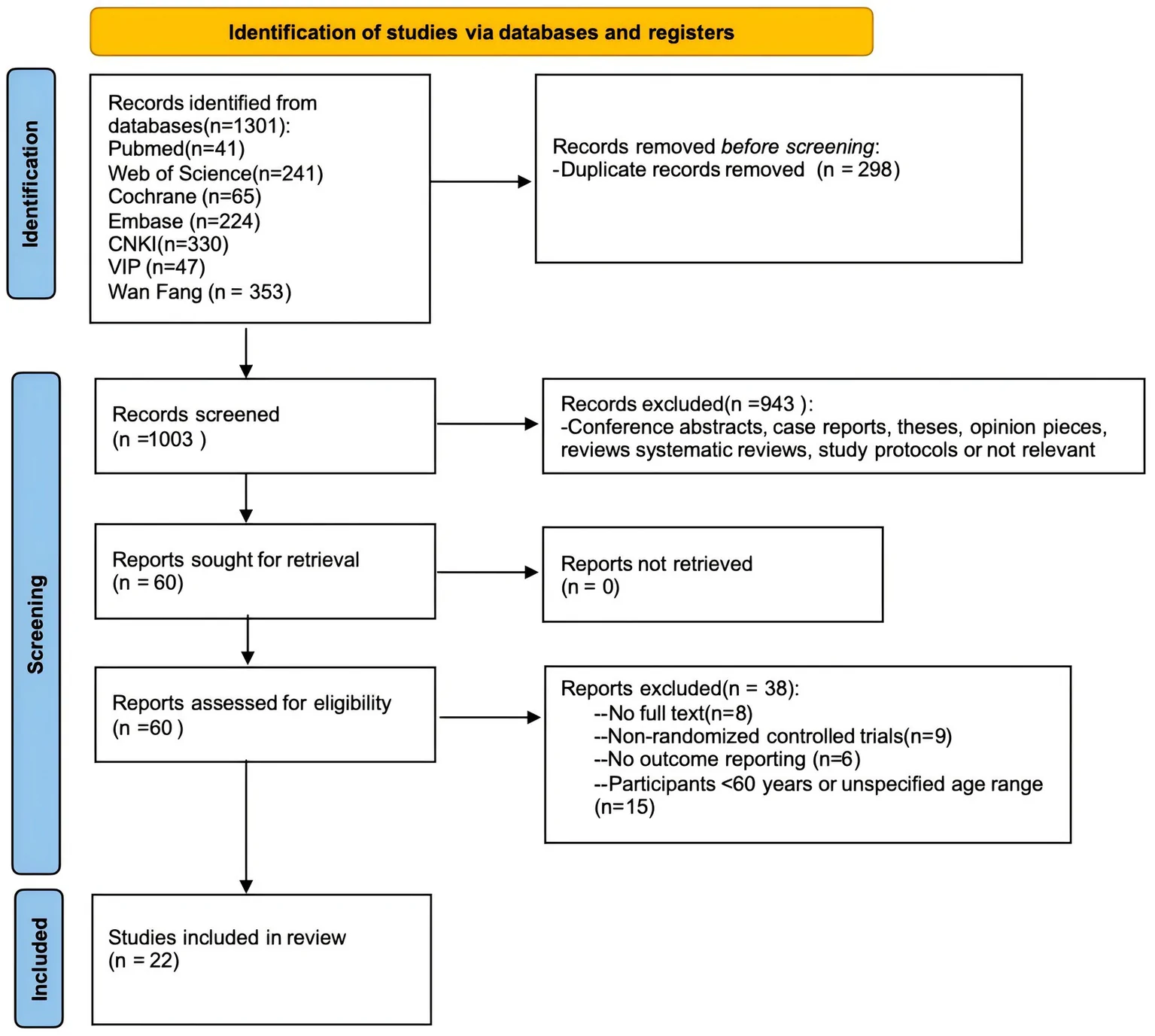
Flow diagram of the study selection process.

The study selection process, conducted in accordance with the PRISMA guidelines, is illustrated in Figure 1. The initial search yielded a total of 1,301 records. After removing duplicates using EndNote (Version X21), 1,003 records remained. Following the screening of titles and abstracts, 943 records were excluded as they were clearly irrelevant to the review topic. Common reasons for exclusion at this stage included: non-randomized controlled trial designs; participants not meeting the age criteria; interventions that were not solely Health Qigong; outcomes not relevant to this review; and publication types such as reviews or conference abstracts. The full texts of the remaining 60 articles were then retrieved and assessed in detail for eligibility. Of these, 38 articles were excluded for the following specific reasons: full text could not be obtained (*n* = 8); the study design was not an RCTs (*n* = 9); participant age was <60 years or the age range was not clearly reported (*n* = 15); and no relevant outcome measures were reported (*n* = 6). Consequently, a total of 22 articles met all inclusion criteria and were included in this systematic review.

### 3.2 Quality of included studies

**Figure 2:**
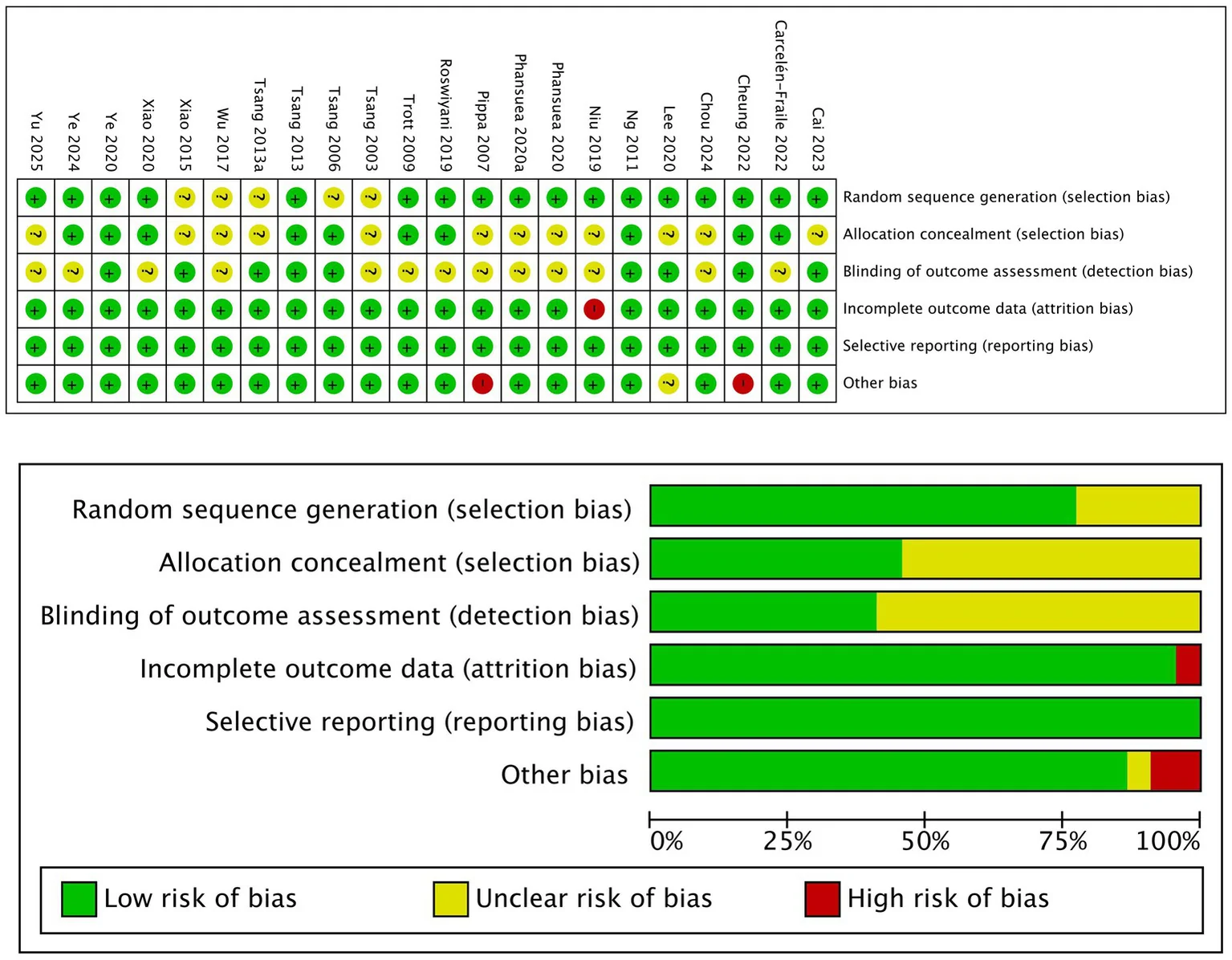
Assessment of risk of bias in the included studies.

The results of the risk of bias assessment for the 22 included randomized controlled trials (RCTs) are summarized in Figure 2. Among the included studies, 17 explicitly reported the method used to generate the randomization sequence. Adequate allocation concealment was described in 10 studies. Blinding of outcome assessors was implemented in 9 studies. Outcome data were complete for 21 studies, and all 22 studies reported all pre-specified outcomes. No other significant sources of bias were identified in 19 of the studies. Overall, based on the assessment, 3 studies were judged to have a high risk of bias, 16 were judged to have an unclear risk of bias, and 3 were judged to have a low risk of bias.

### 3.3 Characteristics of included studies

This analysis included 22 RCTs involving a total of 1,791 participants (19–40). Table 1 presents the characteristics of the included studies. Geographically, 7 studies were conducted in Mainland China (26–28, 31, 34, 39, 40), 7 studies in Hong Kong SAR, China (19, 20, 23–25, 35, 37), 2 studies in Taiwan, China (32, 38), and 2 studies in Thailand (30, 33). The remaining 4 studies originated from Italy (21), Spain (36), Indonesia (29), and Germany (22). While the majority of the evidence originated from East and Southeast Asia, the inclusion of studies from Europe suggested a degree of cross-cultural applicability, providing initial support for the international representativeness and external validity of the findings. Furthermore, only 1 study (22) reported any adverse events related to the Health Qigong intervention, indicating a favorable safety profile for this practice.

**Table 1:** Basic characteristics of included studies.

Author, year, place	Sample size (n), average age (SD) of intervention group and study population	Intervention group	Frequency type (min week)	Mainly outcome indicators	Adverse event
Tsang et al. (2003), Hong Kong (19)	*N* = 50	I: Baduanjin (*n* = 24)	60 min × 2times a week × 12 weeks	GDS; WHOQOL-BREF(HK)	No
	Mean age = 72.90 (9.50)				
		C: traditional remedial rehabilitation (*n* = 26)			
	Chronic physical illnesses				
Tsang et al. (2006), Hong Kong (20)	*N* = 82	I: Baduanjin (*n* = 48)	45 min × 3times a week × 16 weeks	GDS; CGSS; PWI; GHQ	No
	Mean age = 82.11 (7.19)				
		C: newspaper reading (*n* = 34)			
	Depression with chronic physical illnesses				
Pippa et al. (2007), Italy (21)	*N* = 43	I: Qigong (*n* = 22)	90 min × 2 times a week × 16 weeks	6MWT; BMI	No
	Mean age = 68.00 (8.00)	C: daily life (*n* = 21)			
	AF				
von Trott et al. (2009), Germany (22)	*N* = 117	I: Dantian Qigong (*n* = 38)	45 min × 2times a week × 12 weeks	NPAD; SF-36; ADS	5
		C1: exercise therapy (*n* = 39)			
	Mean age = 75.90 (7.60)				
		C2: normal activities (*n* = 40)			
	Recurrent neck pain				
Ng et al. (2011), Hong Kong (23)	*N* = 52	I: Baduanjin (*n* = 23)	15 min × 4times a week × 24 weeks	SF-36;6MWT; MFTE	No
	Mean age = 71.75 (1.05)	C: breathing and walking exercises (*n* = 29)			
	COPD				
Tsang et al. (2013), Hong Kong (24)	*N* = 116	I: Baduanjin and Yijin Jing (*n* = 61)	60 min × 2 times a week × 12 weeks	GDS; LOTCA-G; HGS; TUG;	No
	Mean age = 83.33 (6.30)				
	Frail elders				
		C: newspaper reading (*n* = 55)			
Tsang et al. (2013), Hong kong (25)	*N* = 38	I: Baduanjin (*n* = 21)	45 min × 3times a week × 12 weeks	GDS; CGSS; SQCPD; HGS;5-HT	No
	Mean age = 80.00 (7.00)	C: newspaper reading (*n* = 17)			
	Depression with chronic physical illnesses				
Xiao and Zhuang (2015), China (26).	*N* = 126	I: Liuzijue (*n* = 63)	45 min × 4times a week × 24 weeks	6MWT; MFTE; SF-36	No
	Mean age = 70.90 (1.40)				
		C: walking exercise (*n* = 63)			
	COPD				
Wu et al. (2017), China (27)	*N* = 120	I: Baduanjin (*n* = 60)	30 min × 7 times a week × 4 weeks	MFS; BBS; TUG; SF-36	No
	Mean age = 70.55 (4.26)	C: normal activities (*n* = 60)			
	High risk of fall				
Niu et al. (2019), China (28)	*N* = 60	I: breath Qigong (*n* = 20)	60 min × 7times a week × 12 weeks	LOTCA; MoCA; BI	No
		C1: cognitive training (*n* = 20)			
	Mean age = 66.30 (4.40)				
		C2: Qigong + cognitive training (*n* = 20)			
	VCI				
Roswiyani et al. (2019), Indonesia (29)	*N* = 267	I: wisdom heart Qigong (*n* = 67)	90 min × 2times a week × 8 weeks	WHOQOL-Bref; SWLS; BDI-II; SF-36	No
	Mean age = 73.82 (9.61)				
		C1: art (*n* = 63)			
		C2: integration (*n* = 72)			
	No serious disease				
		C3: normal activities (*n* = 65)			
Phansuea et al. (2020), Thailand (30)	*N* = 66	I: Qigong (*n* = 33)	60 min × 3times a week ×12 weeks	TPSQI	No
	Mean age = 69.00 (6.30)				
		C: Singing and praying activities (*n* = 33)			
	Depression				
Xiao et al. (2020), China (31)	*N* = 85	I: Wuqinxi (*n* = 45)	60 min × 4times a week × 24 weeks	BBS; TUG;6MWT;30SCST; WOMAC	No
	Mean age = 70.70 (9.36)				
		C: lower extremity and aerobic training (*n* = 40)			
	KOA				
Lee et al. (2020), Taiwan (32)	*N* = 30	I: Baduanjin (*n* = 14)	2times a week × 12 weeks	DASS-A; FIM; HGS; PHQ; PWI; TUG;	No
	Mean age >60				
	Depressive symptoms and chronic physical illness				
		C: cognitive training (*n* = 16)			
Phansuea et al. (2020), Thailand (33)	*N* = 66	I: Qigong (*n* = 33)	60 min × 3times a week × 12 weeks	TGDS	No
	Mean age = 69.00 (6.30)				
		C: singing and praying activities (*n* = 33)			
	Depression				
Ye et al. (2020), China (34)	*N* = 56	I: Baduanjin (*n* = 28)	40 min × 3times a week × 12 weeks	COP; WOMAC	No
	Mean age = 65.11 (6.57) KOA	C: normal activities (*n* = 28)			
Cheung et al. (2022), Hong Kong (35)	*N* = 28	I: Baduanjin (*n* = 15)	60 min × 2 times a week × 16 weeks	FFP; SPPB; MBI; GDS; EORTC QLQ-C30	No
	Mean age = 69.20 (3.88)				
		C: light flexibility exercise (*n* = 13)			
	Cancer survivors				
del Carmen Carcelén-Fraile et al. (2022), Spanish (36)	*N* = 117	I: Baduanjin (*n* = 57)	60 min × 2times a week × 12 weeks	PSQI; HADS	No
	Mean age = 69.73 (6.44)				
		C: normal activities (*n* = 60)			
	Postmenopausal women				
Cai et al. (2023), Hong Kong (37)	*N* = 47	I: Baduanjin (*n* = 25)	60 min × 2 times a week × 12 weeks	PWI; FIM; PSQI; TUG; HGS	No
	Mean age = 68.05 (6.59)				
		C: cognitive training and upper arm mobilization (*n* = 22)			
	Depressive				
Chou et al. (2024), Taiwan (38)	*N* = 63	I: Wuqinxi (*n* = 32)	50 min × 3times a week × 12 weeks	BPI; PSQI; BCQ; ATDS	No
	Mean age = 68.81 (3.55)				
		C: normal activities (*n* = 31)			
	Chronic pain and sleep disorders				
Ye et al. (2024), China (39)	*N* = 102	I: Baduanjin (*n* = 51)	60 min × 3times a week × 24 weeks	MoCA; EFS	No
	Mean age = 67.68 (5.19) CF				
		C: normal activities (*n* = 51)			
Yu et al. (2025), China (40)	*N* = 60	I: Baduanjin (*n* = 20)	90 min × 4times a week × 16 weeks	COP	No
	Mean age = 68.25 (7.70)				
		C1: brisk walking (*n* = 20)			
	No serious disease				
		C2: normal activities (*n* = 20)			

SD, Standard deviation; I, intervention group; C, control group; GDS, short-form geriatric depression scale; WHOQOL-BREF(HK), Hong Kong Chinese Version World Health Organization Quality of Life; CGSS, Chinese General Self-efficacy Scale; PWI, Personal Well-Being Index; GHQ, General Health Questionnaire; AF, Chronic atrial fibrillation; 6MWT, 6-min walk test; BMI, body mass index; NPAD, neck pain and disability scale; SF-36, MOS 36-item short-form quality-of-life questionnaire; ADS, Allgemeine Depressions Skala; COPD, chronic obstructive pulmonary disease; MFTE, monitored functional task evaluation; LOTCA-G, Loewenstein Occupational Therapy Cognitive Assessment-Geriatric; HGS, Hand Grip Strength; TUG, timed up and go; SQCPD, self-concept; 5-HT, serum serotonin; MFS, Morse Fall Scale; BBS, Berg Balance Scale; VCI, vascular cognitive impairment; LOTCA, Loewenstein Occupational Therapy Cognitive Assessment; MoCA, Montreal cognitive assessment; BI, Barthel Index; WHOQOL-Bref, World Health Organization Quality of Life; SWLS, The Satisfaction With Life Scale; BDI-II, Beck Depression Inventory-II; KOA, knee osteoarthritis; 30-SCS, 30-s chair stand; WOMAC, Western Ontario and McMaster Universities Osteoarthritis Index; DASS-A, Depression Anxiety Stress Scale-Anxiety; FIM, Functional Independence Measure; PHQ, Patient Health Questionnaire; TGDS, Thai Geriatric Depression Scale; TPSQI, Thai version of the Pittsburgh Sleep Quality Index; COP, Center of pressure; FFP, Fried Frailty Phenotype; SPPB, Short Physical Performance Battery; MBI, Modified Barthel Index; EORTC QLQ-C30, European Organization for Research and Treatment of Cancer Quality of Life Core Questionnaire; PSQI, Pittsburgh Sleep Quality Index; HADS, The Hospital Anxiety and Depression Scale; BPI, Brief Pain Inventory; BCQ, Body Constitution Questionnaire; ATDS, Automatic Tongue Diagnosis System; CF, cognitive frailty; EFS, Edmonton Frail Scale. Phansue et al. (30) and Phansue et al. (33) refer to the same trial but are listed separately for distinct outcome measures; no overlapping data were used in the meta-analysis.

The included trials involved 8 different forms of Health Qigong practice. Specifically, 12 studies employed Baduanjin (19, 20, 23, 25, 27, 32, 34–37, 39, 40), and 2 studies used Wuqinxi (31, 38). One study each combined Baduanjin and Yijinjing (24), or utilized Liu Zi Jue (26), breathing Qigong (28), Dantian Qigong (22), and Wisdom Heart Qigong (29). Three studies did not report the specific form of Qigong used (21, 30, 33).

The study population encompassed a diverse range of older adult groups, including generally healthy individuals, as well as those with depression, depression comorbid with chronic physical illnesses, chronic obstructive pulmonary disease (COPD), knee osteoarthritis (KOA), atrial fibrillation (AF), vascular cognitive impairment (VCI), cognitive frailty (CF) or physical frailty, recurrent neck pain, chronic pain with sleep disorders, high risk of falls, cancer survivors, and postmenopausal women.

Regarding the control group configurations across the 22 studies, 15 employed non-exercise controls, such as maintaining usual daily activities, engaging in singing and praying, receiving cognitive training, reading newspapers, and taking part in art learning (19–21, 24, 25, 27–29, 30, 32–34, 36, 38, 39). In contrast, 5 studies utilized exercise-based controls, including aerobic training, walking programs, and upper limb exercises (23, 26, 31, 35, 37). The remaining 2 studies incorporated both exercise and non-exercise control conditions (22, 40).

Regarding intervention duration, a 12-week protocol was the most frequently employed, utilized in 12 studies (19, 22, 24, 25, 28, 30, 32–34, 36–38). This was followed by 24-week (23, 26, 31, 39) and 16-week (20, 21, 35, 40) protocols, each used in 4 studies. One study implemented an 8-week intervention (29), and 1 study adopted a 4-week protocol (27). Individual session lengths typically ranged from 40 to 60 min. Notably, the intervention frequency across all studies was consistently a minimum of 2 sessions weekly.

The outcome measures examined in this study were categorized into three primary domains: (1) Physical function indicators, which encompassed the 6-min walk test (6MWT), the Timed Up and Go test (TUG), and balance assessments; (2) Psychological and quality of life indicators, consisting of depressive symptoms, overall quality of life (QoL), activities of daily living (ADL), and subjective well-being (SWB); (3) Other indicators, which included cognitive function, pain intensity, and sleep quality.

### 3.4 Meta-analysis of physical function indicators

#### 3.4.1 The 6-minute walk test

**Figure 3:**
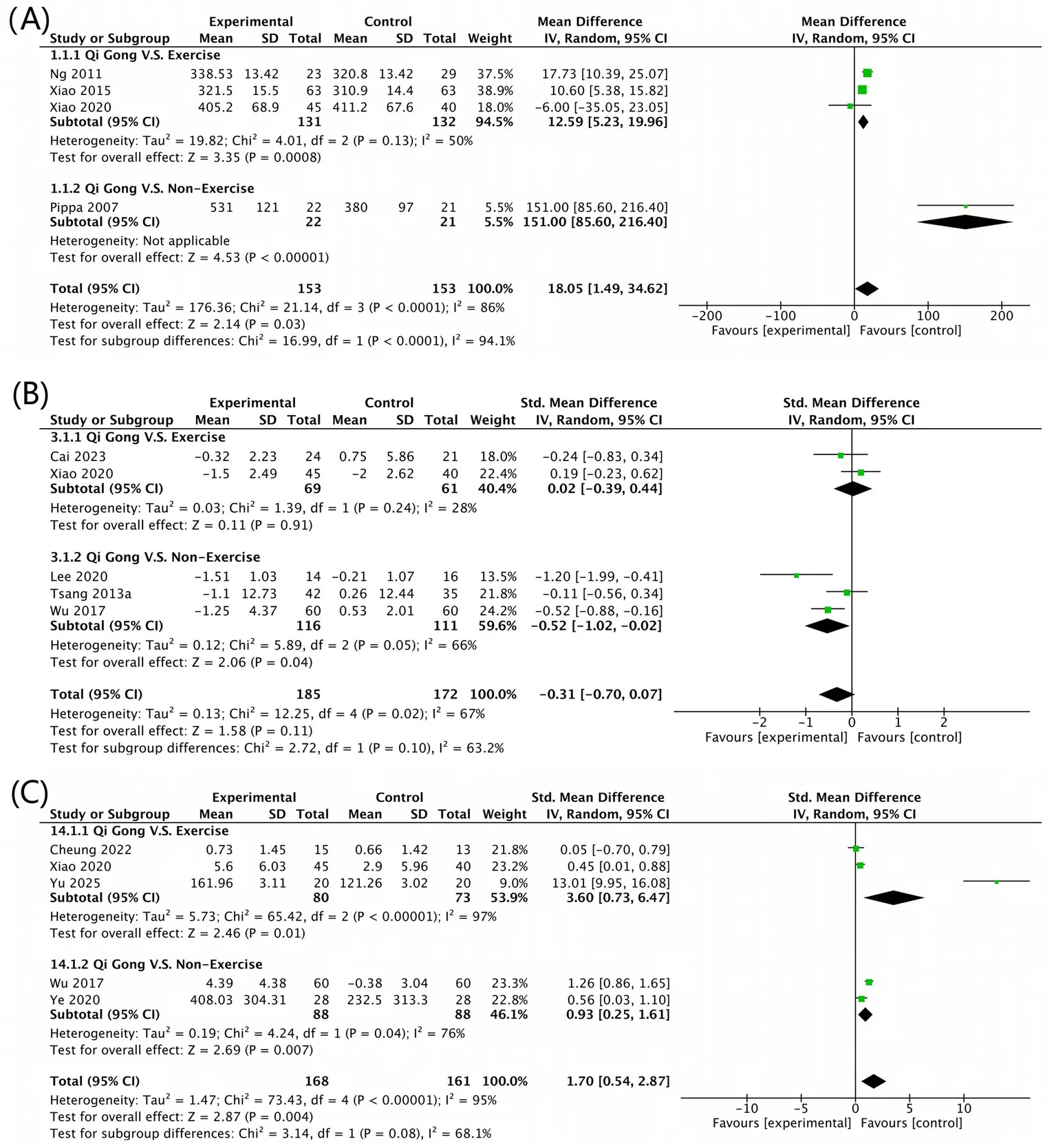
Forest plot of physical function **(A)** 6MWT, **(B)** TUG, **(C)** balance.

In the four included trials (21, 23, 26, 31), the 6MWT was employed as a core outcome measure. A pooled analysis of these trials (n = 306), presented in Figure 3A, indicated that the Health Qigong group demonstrated an increased 6MWT distance compared to the control group (random-effects model: MD = 18.05 m, 95%CI: 1.49–34.62, *p* = 0.03). However, substantial heterogeneity was observed across the studies (*I*^2^ = 86%, *p* < 0.0001). Subgroup analysis based on the type of control intervention revealed that Health Qigong significantly improved the 6MWT distance when compared to both exercise controls (*p* = 0.0008) and non-exercise controls (*p* < 0.00001). To explore the sources of high heterogeneity and assess the robustness of the results, a systematic leave-one-out sensitivity analysis was conducted and presented in Table 2. When the study by Pippa et al. (21) was excluded, the pooled effect remained highly significant (MD = 12.59 m, 95% CI: 5.23–19.96, *p* = 0.0008), and heterogeneity decreased substantially (*I*^2^ = 50%). Conversely, excluding either Ng et al. (23) or Xiao and Zhuang (26) did not resolve the heterogeneity (*I*^2^ fluctuated up to 90%); however, the CIs notably widened and shifted, causing the overall pooled effect to lose statistical significance. Despite these sensitivity fluctuations, when a fixed-effects model was applied to pool all four studies, the overall effect remained highly significant (MD = 13.17 m, 95% CI: 8.96–17.37, *p* < 0.00001).

**Table 2:** Results of sensitive analysis.

Outcomes	Factors (sensitivity analysis)	No. of studies	Pooled effect size (95% CI) and *p* value	Heterogeneity (*I*^2^)
6-Minute walk test	Excluding low-quality study (21)	3	12.59 [5.23, 19.96] (*p* = 0.0008)	*I*^2^ = 50%
	Leave-one-out analysis	3	Stable, except when excluding (23, 26) (result became non-significant)	*I*^2^ = 50% ~ 90% [*I*^2^ = 50% after excluding (21)]
	Fixed-effects model	4	13.17 [8.96, 17.37] (*p* < 0.00001)	*I*^2^ = 86%
Timed up and go test	Excluding low-quality study	/	/	/
	Leave-one-out analysis	4	Stable, except when excluding (31) (result became significant: −0.45 [−0.82, 0.07], *p* = 0.02)	*I*^2^ = 52% ~ 74% [*I*^2^ = 52% after excluding (31)]
	Fixed-effects model	5	−0.27 [−0.48, −0.06] (*p* = 0.01)	*I*^2^ = 67%
Balance	Excluding low-quality study (35)	4	2.27 [0.87, 3.68] (*p* < 0.00001)	*I*^2^ = 96%
	Leave-one-out analysis	4	Stable	*I*^2^ = 75% ~ 96% [*I*^2^ = 75% after excluding (40)]
	Fixed-effects model	5	0.81 [0.57, 1.05] (*p* < 0.00001)	*I*^2^ = 95%
Depression	Excluding low-quality study (35)	9	−0.68 [−1.15, −0.21] (*p* = 0.005)	*I*^2^ = 89%
	Leave-one-out analysis	9	Stable	*I*^2^ = 65% ~ 90% [*I*^2^ = 64% after excluding (33)]
	Fixed-effects model	10	−0.43 [−0.58, −0.28] (*p* < 0.00001)	*I*^2^ = 87%
QoL	Excluding low-quality study (35)	6	0.86 [0.10, 1.62] (*p* = 0.03)	*I*^2^ = 94%
	Excluding low-quality study—exercise subgroup (35)	2	2.13 [1.56, 2.71] (*p* < 0.00001)	*I*^2^ = 48%
	Leave-one-out analysis	6	Stable, except when excluding (23, 26, 27) (result became non-significant)	*I*^2^ = 89% ~ 94%
	Fixed-effects model	7	0.63 [0.46, 0.80] (*p* < 0.00001)	*I*^2^ = 93%
ADL	Excluding low-quality studies (28, 35)	2	0.27 [−0.19, 0.74] (*p* = 0.25)	*I*^2^ = 0%
	Leave-one-out analysis	3	Stable	*I*^2^ = 0% ~ 24% [*I*^2^ = 24% after excluding (35)]
	Random-effects model	4	0.01 [−0.33, 0.34] (*p* = 0.97)	*I*^2^ = 0%
SWB	Excluding low-quality study	/	/	/
	Leave-one-out analysis	2	Stable	*I*^2^ = 85% ~ 96%
	Fixed-effects model	3	12.04 [9.22, 14.87] (*p* < 0.00001)	*I*^2^ = 92%
Cognitive function	Excluding low-quality study (28)	2	0.52 [0.24, 0.79] (*p* = 0.0002)	*I*^2^ = 0%
	Leave-one-out analysis	2	Stable	*I*^2^ = 0%
	Random-effects model	3	0.48 [0.23, 0.72] (*p* = 0.0002)	*I*^2^ = 0%
Pain	Excluding low-quality study	/	/	/
	Leave-one-out analysis	3	Stable, except when excluding (31, 38, 39), (result became non-significant)	/
	Random-effects model	4	−0.26 [−0.50, −0.03] (*p* = 0.03)	*I*^2^ = 0%
Sleep quality	Excluding low-quality study (30)	2	−0.39 [−0.66, −0.12] (*p* = 0.005)	*I*^2^ = 2%
	Leave-one-out analysis	3	Stable, except when excluding (36) (result became non-significant)	*I*^2^ = 2% ~ 69% [*I*^2^ = 2% after excluding (30)]
	Fixed-effects model	4	−0.52 [−0.75, −0.28] (*p* < 0.0001)	*I*^2^ = 54%

#### 3.4.2 The timed up and go test

A total of five trials included in this study reported TUG outcomes (24, 27, 31, 32, 37). A meta-analysis of these trials (*n* = 357), presented in Figure 3B, revealed that the Health Qigong group tended to complete the TUG in less time than the control group; however, this difference did not reach statistical significance (random-effects model: SMD = −0.31, 95% CI: −0.70 to 0.07, *p* = 0.11). Moderate heterogeneity was observed among the studies (*I*^2^ = 67%, *p* = 0.02). Subgroup analyses indicated that Health Qigong interventions yielded a significant improvement compared to non-exercise control groups (*p* = 0.04), whereas no statistically significant difference was found when compared to exercise control groups (*p* = 0.91). To assess the stability of the results, we performed sensitivity analyses. Leave-one-out analysis showed that the pooled result was sensitive; after excluding the study by Xiao et al. (31), the overall pooled effect changed from non-significant to statistically significant (p = 0.02), and heterogeneity decreased (*I*^2^ = 52%). Moreover, when the analysis model was changed from random-effects to fixed-effects, the overall effect also became highly statistically significant (SMD = -0.27, 95% CI: −0.48 to −0.06, *p* = 0.01).

#### 3.4.3 Balance

A total of five randomized controlled trials evaluated the effects of Health Qigong on balance ability in older adults (27, 31, 34, 35, 40). A meta-analysis of these studies (*n* = 329), presented in Figure 3C, demonstrated that the Health Qigong group exhibited significant improvement in balance ability compared to control groups (random-effects model: SMD = 1.7, 95% CI: 0.54 to 2.87, *p* = 0.004). However, substantial heterogeneity was observed across the studies (*I*^2^ = 95%). Subgroup analyses indicated that Health Qigong yielded significant improvements regardless of whether the comparator was an exercise or non-exercise control group. Further sensitivity analysis revealed that excluding the study by Yu et al. (40) reduced the heterogeneity (*I*^2^ = 75%), suggesting that this study was a primary source of the high heterogeneity.

### 3.5 Meta-analysis of psychological and quality of life indicators

#### 3.5.1 Depression

**Figure 4:**
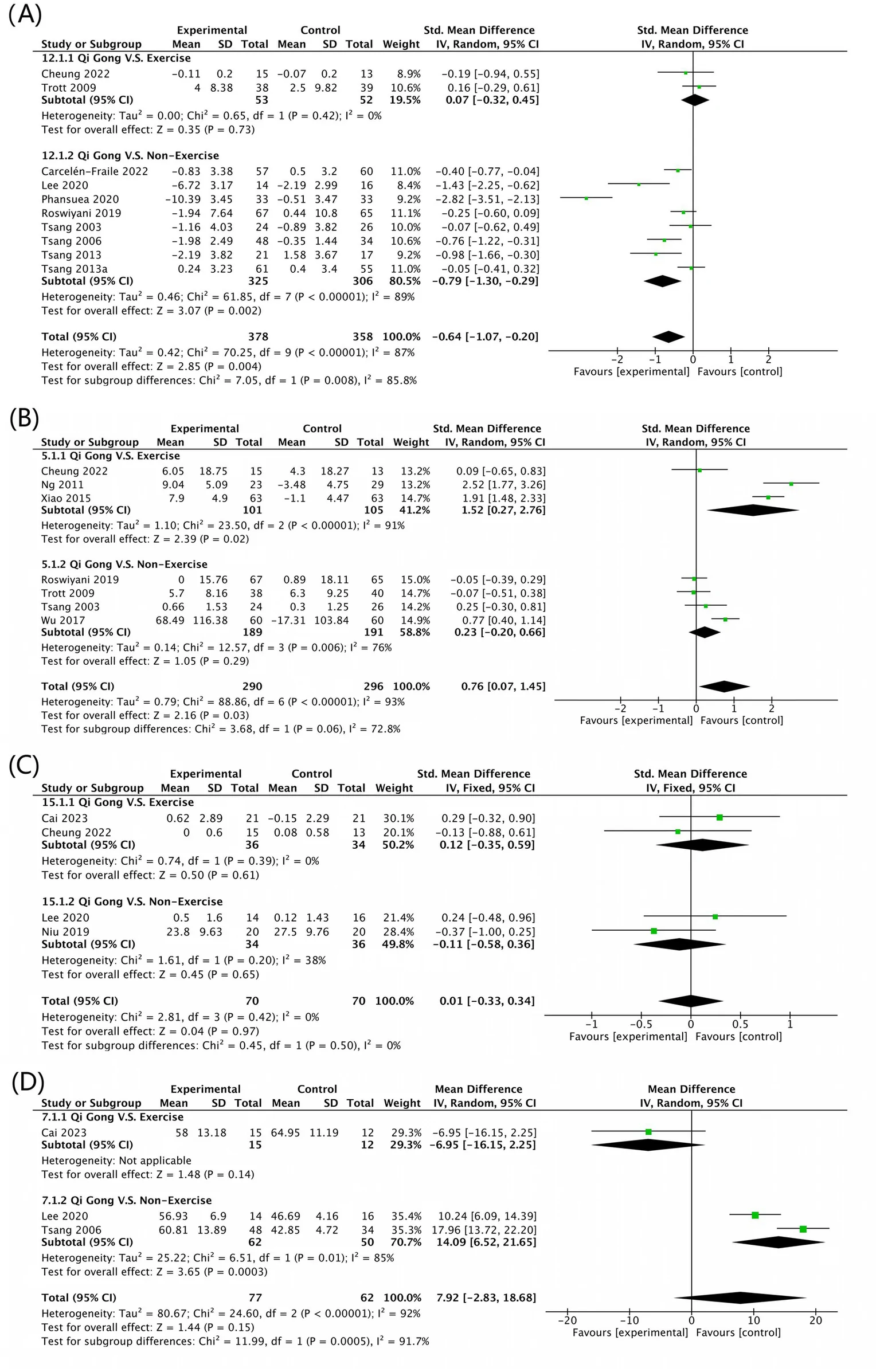
Forest plot of psychology and quality of life: **(A)** depression, **(B)** QoL, **(C)** ADL, and **(D)** SWB.

A total of 10 randomized controlled trials were included in this analysis (19, 20, 22, 24, 25, 29, 32, 33, 35, 36). The meta-analysis results (*n* = 736), presented in Figure 4A, demonstrated that the Health Qigong group was superior to the control group in improving depressive symptoms in older adults (random-effects model: SMD = −0.64, 95% CI: −1.07 ~ −0.20, *p* = 0.004). High heterogeneity was observed across the studies (*I*^2^ = 87%). Subgroup analysis suggested that the type of control intervention was a potential source of this heterogeneity (*I*^2^ = 85.8%, *p* = 0.008); Health Qigong demonstrated a significant antidepressant effect compared to non-exercise control groups (*p* = 0.002), whereas no statistically significant difference was found when compared to exercise control groups (*p* = 0.73). Further sensitivity analysis revealed that neither excluding low-quality studies nor applying a fixed-effect model altered the significance of the overall effect. Leave-one-out analysis indicated that, while the overall conclusion remained robust, the exclusion of the study by Phansuea et al. (33) substantially reduced the heterogeneity (*I*^2^ = 64%), indicating that this study may have been the primary contributor to the observed heterogeneity.

#### 3.5.2 Quality of life

A total of seven trials were included in this analysis (19, 22, 23, 26, 27, 29, 35). The meta-analysis (*n* = 586), presented in Figure 4B, demonstrated that the overall effect of Health Qigong on QoL reached statistical significance (random-effects model: SMD = 0.76, 95% CI: 0.07 to 1.45, *p* = 0.03). Notably, high heterogeneity was observed across the studies (*I*^2^ = 93%). Subgroup analysis indicated that Health Qigong significantly improved QoL in studies with exercise control groups (*p* = 0.02); however, heterogeneity within this subgroup remained substantial (*I*^2^ = 91%). In contrast, no significant effect was detected in studies with non-exercise control groups (*p* = 0.29). Sensitivity analysis indicated some instability in the pooled results. Leave-one-out analysis revealed that the overall effect lost statistical significance when the studies by Ng et al. (23), Xiao and Zhuang (26), or Wu et al. (27) were individually removed. Furthermore, within the active-control subgroup, excluding the study by Cheung et al. (35), which had a higher risk of bias, substantially reduced subgroup heterogeneity (*I*^2^ = 48%) and yielded a more significant pooled effect (*p* < 0.00001); however, this did not appreciably lower the overall heterogeneity (*I*^2^ = 94%).

#### 3.5.3 Activities of daily living

A total of four trials were included in this analysis [(28, 32, 35, 37), presented in Figure 4C]. The pooled analysis (*n* = 140) demonstrated that Health Qigong did not produce a significant effect on improving ADL in older adults (fixed-effects model: SMD = 0.01, 95% CI: −0.33 to 0.34, *p* = 0.97), with no heterogeneity observed across the studies (*I*^2^ = 0%). Subgroup analyses further revealed that Health Qigong showed no statistically significant improvement in ADL, regardless of whether the control intervention was exercise-based or non-exercise-based. Sensitivity analyses confirmed the robustness of these findings.

#### 3.5.4 Subjective well-being

A total of three trials reported SWB outcomes using the Personal Well-Being Index (PWI) (20, 32, 37). A meta-analysis of these studies (n = 139), presented in Figure 4D, revealed no significant difference between the Health Qigong and control groups (random-effects model: MD = 7.92 points, 95% CI: −2.83 to 18.68, *p* = 0.15), with very high heterogeneity observed (*I*^2^ = 92%). Subgroup analysis indicated that Health Qigong significantly improved SWB compared to non-exercise control groups (MD = 14.09 points, *p* = 0.0003), yet showed no advantage and even a slight negative trend when compared with conventional exercise controls (MD = −6.95 points, *p* = 0.14). Sensitivity analysis indicated that the overall effect direction remained relatively stable upon leave-one-out analysis. However, re-fitting the data with a fixed-effect model would have yielded a highly significant overall effect (MD = 12.04 points, *p* < 0.00001). Given the extreme heterogeneity observed for this outcome (*I*^2^ = 92%), the more conservative estimate from the random-effects model is statistically appropriate.

### 3.6 Meta-analysis of other related indicators

#### 3.6.1 Cognition

**Figure 5:**
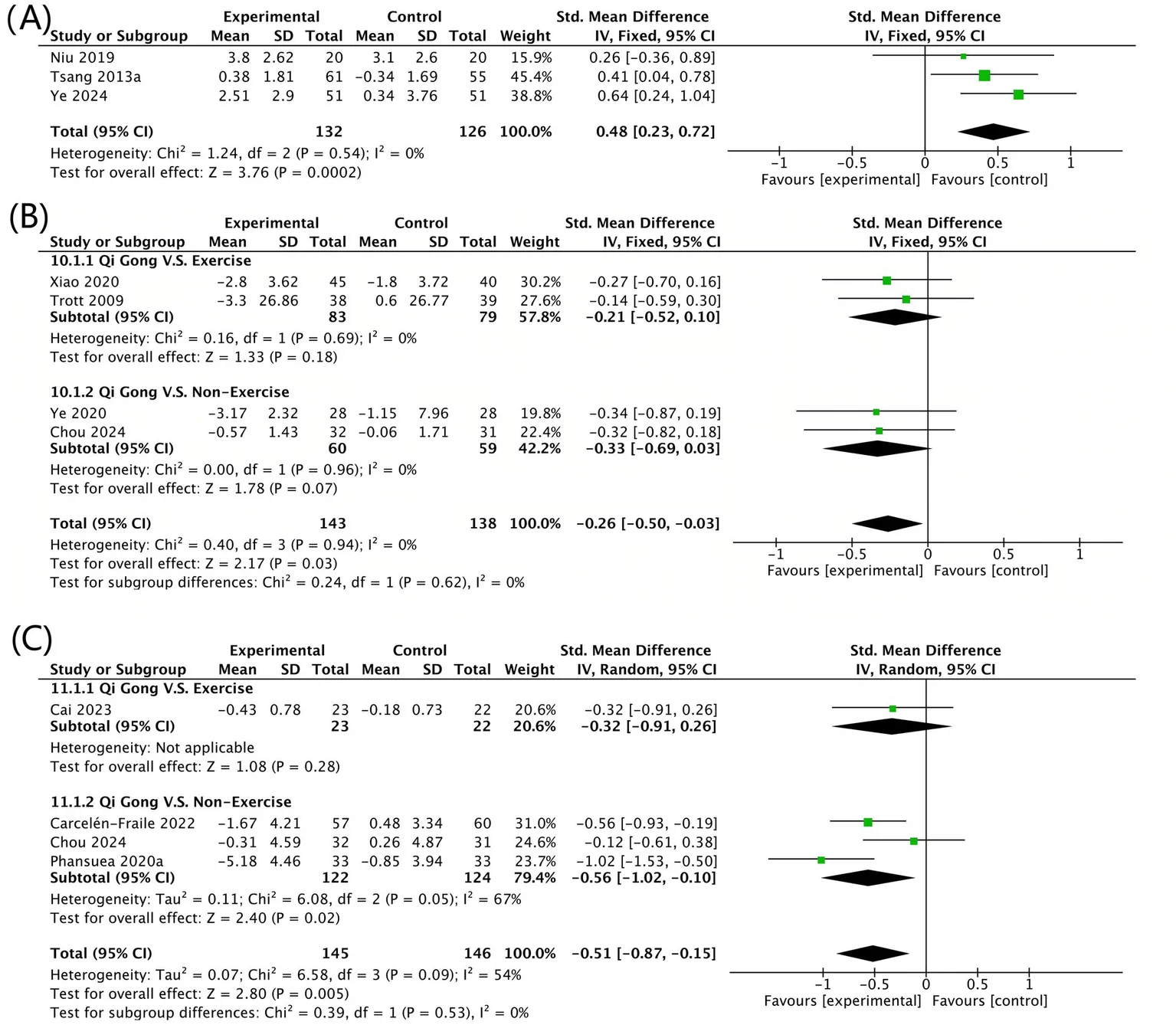
Forest plot of other indicators: **(A)** cognition, **(B)** pain, **(C)** sleep quality.

A total of three trials were included in this analysis (24, 28, 39). The meta-analysis (*n* = 258), presented in Figure 5A, demonstrated that the Health Qigong group was significantly superior to the control group in improving cognitive function (fixed-effects model: SMD = 0.48, 95% CI: 0.23 to 0.72, *p* = 0.0002), with no heterogeneity observed across the studies (*I*^2^ = 0%). Sensitivity analyses further supported the robustness of this finding.

#### 3.6.2 Pain

A total of four trials were included in this analysis (22, 31, 34, 38). The meta-analysis (*n* = 281), presented in Figure 5B, demonstrated that pain scores were significantly lower in the Health Qigong group compared to the control group (fixed-effects model: SMD = −0.26, 95% CI: −0.50 to −0.03, *p* = 0.03), with no heterogeneity across studies (*I*^2^ = 0%). However, sensitivity analysis indicated that this result had limited robustness. Although the random-effects model yielded a consistent finding (*p* = 0.03), leave-one-out analysis revealed that the statistical significance was lost when any one of the studies by Xiao et al. (31), Chou et al. (38), or Ye et al. (34) was removed. This suggests that the pooled conclusion regarding the pain-relieving effect of Health Qigong is heavily influenced by individual studies and should be interpreted with caution.

#### 3.6.3 Sleep quality

A total of four RCTs were included in this analysis (30, 36–38). The meta-analysis (*n* = 291), presented in Figure 5C, demonstrated that Health Qigong significantly improved sleep quality in older adults (random-effects model: SMD = −0.51, 95% CI: −0.87 to −0.15, *p* = 0.005), with moderate heterogeneity observed across the studies (*I*^2^ = 54%). Sensitivity analysis revealed that excluding the low-quality study by Phansuea et al. (33) substantially reduced the heterogeneity (*I*^2^ = 2%), indicating that this study was the primary source of the observed heterogeneity. However, leave-one-out analysis also showed that the removal of del Carcelén-Fraile et al. (36) caused the overall statistical significance to disappear, suggesting that this high-weight study played a crucial role in supporting the overall effect estimate.

### 3.7 Publication bias

Egger’s linear regression test was conducted to assess publication bias across all outcome measures. The resulting *p*-values were 0.076, 0.486, 0.212, 0.083, 0.798, 0.996, 0.362, 0.650, 0.361, and 0.885, respectively. Using a significance level of *p* < 0.05, none of these results reached statistical significance, indicating that no substantial publication bias was detected in this meta-analysis.

### 3.8 Subgroup analyses

The results of subgroup analyses for depression and QoL are presented in Table 3, revealing distinct response patterns to Health Qigong intervention characteristics between the two outcomes.

**Table 3:** Results of subgroup analyses for depression and quality of life.

Outcome	Factors	Subgroups	No. of studies	Pooled SMD (95% CI)	*I*^2^ (*p*-value)	Subgroup difference
Depression	Quality	Low	1	−0.19 [−0.94, 0.55]	*p* = 0.61	*I*^2^ = 14.9% (*p* = 0.28)
		Medium and high	9	−0.68 [−1.15, −0.21]	*I*^2^ = 89% (*p* = 0.005)	
	Region	Asian	8	−0.79 [−1.34, −0.23]	*I*^2^ = 89% (*p* = 0.006)	*I*^2^ = 62.3% (*p* = 0.10)
		Non-Asian	2	−0.13 [−0.69, 0.42]	*I*^2^ = 73% (*p* = 0.63)	
	Intervention type	Baduanjin	6	−0.59 [−0.94, −0.24]	*I*^2^ = 56% (*p* = 0.0009)	*I*^2^ = 0% (*p* = 0.84)
		Other Qigong	4	−0.70 [−1.64, 0.25]	*I*^2^ = 95% (*p* = 0.15)	
	Session duration	≥60 min	6	−0.60 [−1.21, 0.02]	*I*^2^ = 91% (*p* = 0.06)	*I*^2^ = 0% (*p* = 0.84)
		<60 min	3	−0.50 [−1.21, 0.21]	*I*^2^ = 82% (*p* = 0.17)	
	Frequency	≥3 times/week	3	−1.51 [−2.73, −0.28]	*I*^2^ = 92% (*p* = 0.02)	*I*^2^ = 74.7% (*p* = 0.05)
		<3times/week	7	−0.24 [−0.51, 0.03]	*I*^2^ = 55% (*p* = 0.08)	
	Total duration	>12 weeks	2	−0.56 [−1.09, −0.02]	*I*^2^ = 38% (*p* = 0.04)	*I*^2^ = 8.8% (*p* = 0.33)
		=12 weeks	7	−0.76 [−1.40, −0.11]	*I*^2^ = 91% (*p* = 0.02)	
		<12 weeks	1	−0.25 [−0.60, 0.09]	*p* = 0.15	
	Control group type	Exercise control	2	0.07 [−0.32, 0.45]	*I*^2^ = 0% (*p* = 0.73)	*I*^2^ = 85.8% (*p* = 0.008)
		Non-exercise control	8	−0.79 [−1.30, −0.29]	*I*^2^ = 89% (*p* = 0.002)	
QoL	Quality	Low	1	0.09 [−0.65, 0.83]	*p* = 0.81	*I*^2^ = 50.4% (*p* = 0.16)
		Medium and high	6	0.86 [0.10, 1.62]	*I*^2^ = 94% (*p* = 0.03)	
	Region	Asian	6	0.90 [0.13, 1.68]	*I*^2^ = 94% (*p* = 0.02)	*I*^2^ = 78% (*p* = 0.03)
		Non-Asian	1	−0.07 [−0.51, 0.38]	*p* = 0.76	
	Intervention type	Baduanjin	4	0.89 [0.02, 1.77]	*I*^2^ = 89% (*p* = 0.05)	*I*^2^ = 0% (*p* = 0.70)
		Other Qigong	3	0.59 [−0.66, 1.85]	*I*^2^ = 97% (*p* = 0.35)	
	Session duration	≥60 min	3	0.04 [−0.23, 0.31]	*I*^2^ = 0% (*p* = 0.77)	*I*^2^ = 80.5% (*p* = 0.02)
		<60 min	4	1.26 [0.24, 2.27]	*I*^2^ = 95% (*p* = 0.02)	
	Frequency	≥3 times/week	3	1.70 [0.71, 2.68]	*I*^2^ = 92% (*p* = 0.0008)	*I*^2^ = 90.5% (*p* = 0.001)
		<3 times/week	4	0.01 [−0.22, 0.24]	*I*^2^ = 93% (*p* = 0.93)	
	Total duration	>12 weeks	3	1.52 [0.27, 2.76]	*I*^2^ = 91% (*p* = 0.02)	*I*^2^ = 60.8% (*p* = 0.08)
		=12 weeks	2	0.06 [−0.29, 0.40]	*I*^2^ = 0% (*p* = 0.75)	
		<12 weeks	2	0.36 [−0.45, 1.17]	*I*^2^ = 90% (*p* = 0.39)	
	Control group type	Exercise control	3	1.52 [0.27, 2.76]	*I*^2^ = 91% (*p* = 0.02)	*I*^2^ = 72.8% (*p* = 0.06) [excluding (35) *I*^2^ = 96.3% (*p* < 0.00001)]
		Non-exercise control	4	0.23 [−0.20, 0.66]	*I*^2^ = 76% (*p* = 0.29)	

For depression, between-subgroup differences were primarily driven by control group type (*p* = 0.008) and practice frequency (*p* = 0.05). Within-subgroup analyses indicated that the antidepressant effect of Health Qigong was concentrated in comparisons with non-exercise controls (SMD = −0.79, 95% CI: −1.30 to −0.29) and in high-frequency practice (≥3 sessions/week; SMD = −1.51, 95% CI: −2.73 to −0.28), whereas the effects did not reach statistical significance in active-control and low-frequency (<3 sessions/week) subgroups. Notably, consistent benefits for depression were observed in subgroups of moderate- to high-quality studies, Asian populations, Baduanjin Qigong, and longer intervention durations (≥12 weeks). In contrast, session duration—whether ≥60 min or not—showed no significant effect.

For QoL, between-subgroup differences were more widespread, involving geographic region (*p* = 0.03), session duration (*p* = 0.02), and practice frequency (*p* = 0.001). After excluding one low-quality study, the difference by control type also reached high significance (*p* < 0.000001). Within-subgroup analysis revealed strict boundary conditions for Health Qigong’s significant effect on QoL: benefits were evident in Asian populations (SMD = 0.90, 95% CI: 0.13 to 1.68), sessions lasting <60 min (SMD = 1.26, 95% CI: 0.24 to 2.27), high-frequency practice (≥3 sessions/week; SMD = 1.70, 95% CI: 0.71 to 2.68), and longer interventions (>12 weeks; SMD = 1.52, 95% CI: 0.27–2.76). In contrast, Health Qigong did not significantly improve QoL in non-Asian regions, other Health Qigong types, sessions ≥60 min, low-frequency practice (<3 sessions/week), short-duration interventions (≤12 weeks), or comparisons with non-exercise controls.

## 4 Discussion

This systematic review evaluated the effects of Health Qigong on quality of life and physical and mental health outcomes in older adults, incorporating 22 RCTs comprising 1,791 participants. Overall, Health Qigong appears to confer tangible benefits for physical function (6MWT and balance), mental health (depressive symptoms), and QoL, with promising trends also observed for common geriatric conditions such as cognitive function, pain, and sleep disturbances. However, improvements in ADL did not reach statistical significance, and the enhancement of SWB was largely confined to comparisons with non-exercise controls. Subgroup and sensitivity analyses further delineated the potential conditions under which Health Qigong is effective and the boundaries of its clinical application.

### 4.1 Positioning and limitations of Health Qigong in improving physical function

This study indicates that Health Qigong may meaningfully improve 6MWT performance and balance in older adults; however, it offers no additional benefit over conventional exercise for the TUG. This suggests that, as a low-intensity, low-impact mind–body intervention, the core value of Health Qigong may lie not in providing a stronger physical stimulus than standard training, but rather in offering a safe alternative for older adults with limited physical capacity or those unable to tolerate moderate- to high-intensity exercise.

This clinical positioning becomes particularly clear when viewed alongside comparable interventions. Existing evidence suggests that Tai Chi tends to outperform conventional exercise in reducing TUG time and improving several balance measures among healthy older adults (41). This may not indicate an absolute superiority of one modality over the other, but rather point to their differential suitability for specific populations and clinical scenarios. Tai Chi involves substantial weight shifting and single-leg stance, placing relatively higher demands on lower-limb strength and hand–eye coordination (42); in contrast, Health Qigong primarily consists of bilaterally symmetrical, repetitive movements with limited spatial displacement and a lower entry threshold (43). Thus, Tai Chi may be better suited to healthy older adults with relatively preserved physical function, whereas Health Qigong may be more accessible for frail or exercise-naïve older individuals, offering a higher safety margin and greater ease of community implementation and long-term adherence.

Importantly, the lack of an overall advantage for Health Qigong in the TUG analysis is likely obscured by data from specific pathological subgroups. Sensitivity analyses showed that the overall effect of Health Qigong on TUG became significant when the KOA study was removed or a fixed-effect model was applied (31). This may be because the sit-to-stand and turning components of the TUG impose mechanical stress on degenerated cartilage, and Health Qigong appears unable to overcome the localized physical and pain-related limitations in KOA patients over the short term. Conversely, for frail older adults whose limitations primarily stem from diminished neuromuscular control without major structural joint damage, Health Qigong may effectively activate their motor potential.

This further suggests that the benefits of Health Qigong may be constrained by pathological boundaries: meaningful improvements in dynamic mobility are more likely among older adults whose core skeletal structures, such as joints, remain free of severe organic pathology. Similarly, the heterogeneity in balance outcomes dropped substantially after removing the study on healthy older adults, which conversely supports the notion that intact joints and neural pathways may be a prerequisite for Health Qigong to translate into balance gains (40). Moreover, the 6MWT results were heavily influenced by the COPD study, highlighting the targeted rehabilitative value of deep abdominal breathing for individuals with cardiopulmonary limitations.

Therefore, in clinical practice, Health Qigong exercise prescription should be individualized based on the patient’s baseline health status. For community-dwelling older adults who are healthy or only in the pre-frailty stage, Health Qigong can serve as a public health intervention for fall prevention and maintenance of physical capacity. For patients with degenerative conditions such as KOA, Health Qigong is appropriate only as an adjunct strategy, and deep knee flexion movements should be avoided. For COPD patients, the breath regulation component of Health Qigong should be emphasized.

### 4.2 Mechanisms of Health Qigong’s benefits for mental health and quality of life

Regarding mental health, this study confirms that Health Qigong may have meaningful potential to alleviate depressive symptoms in older adults. However, subgroup analysis revealed that this antidepressant effect did not demonstrate a clear advantage over conventional exercise. This suggests that the mood-regulating benefits of Health Qigong may primarily arise from non-specific physiological responses common to low-to-moderate intensity aerobic exercise or from the social support gained through group-based practice, rather than from the unique “qi” or “mind-intent” components of the practice.

In contrast, for multidimensional QoL encompassing physical, psychological, and social functioning Health Qigong exhibited a trend toward superiority over conventional exercise. This may be because conventional exercise mainly targets physical adaptations of the musculoskeletal and cardiorespiratory systems, whereas Health Qigong, through the synergistic regulation of body (posture), breath, and mind, establishes a distinctive “body–mind co-regulation” mechanism (5). This deep integration of physical movement with meditative introspection may more effectively activate the parasympathetic nervous system and counteract chronic stress, thereby producing broader psychosomatic resonance (44). It should be noted that the pathways to benefit may differ by baseline health status: improvements in QoL for healthy older adults may stem more from psychological enrichment, while for those with chronic diseases, relief from somatic symptoms may play a more central role.

As for the lack of significant improvement in ADL, this may be attributable to a ceiling effect commonly encountered in ADL assessments (45). The majority of participants in this review were older adults with basic functional independence, leaving limited room for improvement on standard measures such as the Barthel Index or FIM. Therefore, future studies should consider adopting the more sensitive Instrumental Activities of Daily Living (IADL) scale to capture the potential benefits of Health Qigong on more complex social functioning.

### 4.3 Dose–response thresholds and cross-cultural responses

Subgroup analyses further delineated the dose thresholds for Health Qigong’s effects on mental health and QoL. For depression, significant benefits were primarily concentrated in studies employing high-frequency (≥3 sessions/week) and long-duration (≥12 weeks) protocols, suggesting that the re-establishment of emotional rhythms may depend on sustained stimulation. For QoL, the conditions for efficacy appeared even more stringent, observed only in the combination of high frequency, short session duration, and long intervention period (≥3 sessions/week, <60 min/session, >12 weeks). This indicates that overly long single sessions may easily lead to physical exhaustion in older adults; moderate, regular practice is more likely to translate physiological improvements into enhanced QoL.

Moreover, the benefits of Health Qigong for depression and QoL have yet to reach statistical significance in non-Asian populations. This may be attributable to cultural familiarity: as a traditional Eastern mind–body practice, Health Qigong enjoys inherent cultural acceptance among Asian populations, which may help lower beginners’ psychological resistance and more readily induce deep relaxation and positive intervention expectations (46). However, a critical and objective limitation must not be overlooked—primary studies conducted in non-Asian regions remain extremely scarce (only 1–2 per outcome), most likely resulting in insufficient statistical power. Therefore, it cannot be concluded that Health Qigong has geographical limitations; future cross-cultural, large-sample trials are urgently needed to verify these findings.

### 4.4 Limitations

This study has several limitations. First, the methodological quality of the included studies was uneven, with some lacking adequate details on randomization and blinding procedures. Second, sample sizes were generally small, and substantial heterogeneity was observed across Health Qigong styles and measurement instruments. Third, most primary studies did not comprehensively report key intervention parameters such as exercise intensity, which constrained further exploration and precise evaluation of Health Qigong prescription components. Fourth, the study population ranged from healthy individuals to patients with chronic diseases; this clinical heterogeneity may limit the generalizability of the overall findings. Fifth, the geographic distribution of evidence was imbalanced, with a scarcity of data from non-Asian populations, warranting caution in cross-cultural extrapolation.

## 5 Conclusion

Current evidence suggests that Health Qigong may serve as a potential non-pharmacological intervention for promoting multidimensional health and supporting healthy aging in older adults. Compared with non-exercise interventions, Qigong may yield benefits in physical function, psychological well-being, and overall quality of life; compared with conventional exercise, it demonstrates largely comparable effects on most outcomes, yet may offer unique advantages in enhancing QoL and dynamic balance. In terms of intervention strategy, optimal psychosomatic benefits appear to be achieved with a regimen characterized by relatively high frequency (≥3 sessions/week), long duration (≥12 weeks), and moderate single-session length (<60 min). Moreover, the specific benefits of Qigong appear to be modulated by individuals’ baseline pathological status; thus, clinical application should entail individualized assessment and prescription modification according to the presence of organic or degenerative conditions. Given the current scarcity of evidence from non-Asian regions and insufficient statistical power, the generalizability of Qigong requires further validation across broader geographic and cultural contexts. Future implementation should be carefully tailored to individuals’ baseline health status and psychosocial characteristics to maximize benefits.

## Data Availability

The original contributions presented in the study are included in the article/Supplementary material, further inquiries can be directed to the corresponding authors.
