# Supplementary material for: Effects of Health Qigong on quality of life, physical function, and mental health in older adults: a systematic review and meta-analysis of randomized controlled trials
Source: Front Public Health. 2026 Aug 26;14:1916466. doi: 10.3389/fpubh.2026.1916466 (PMC13541517; doi:10.3389/fpubh.2026.1916466)
Supplement: Supplementary file 1 [file Table_1.DOCX]

Supplementary Material

**Table S1. Search strategies for English and Chinese databases**

| Database | Search strategy | Notes / limits |
| --- | --- | --- |
| PubMed | ( "Qigong"[Mesh] OR "Qi Gong"[Title/Abstract] OR "Ch'i Kung"[Title/Abstract] OR "Chi Kung"[Title/Abstract] OR "Qigong"[Title/Abstract] ) AND ( "Aged"[Mesh] OR "Aging"[Mesh] OR "Elderly"[Title/Abstract] OR "Older Adults"[Title/Abstract] OR "Aged"[Title/Abstract] OR "Senior"[Title/Abstract] ) AND ( "Health Status"[Mesh] OR "Health"[Mesh] OR "Quality of Life"[Mesh] OR "Physical Health"[Title/Abstract] OR "Mental Health"[Title/Abstract] OR "Health Status"[Title/Abstract] OR "Overall Health"[Title/Abstract] OR "Well-being"[Title/Abstract] ) AND ( "Randomized Controlled Trial"[Publication Type] OR "Randomized Controlled Trial"[Title/Abstract] OR "RCT"[Title/Abstract] OR "Controlled Clinical Trial"[Publication Type] ) | MeSH + title/abstract; RCT filter; from inception to May 2025 |
| Cochrane Library | #1 MeSH descriptor: [Qigong] explode all trees #2 (Qi Gong OR Ch'i Kung OR Chi Kung):ti,ab,kw #3 #1 OR #2 #4 MeSH descriptor: [Aged] explode all trees #5 (elderly OR older adult* OR aged):ti,ab,kw #6 #4 OR #5 #7 MeSH descriptor: [Health Status] explode all trees #8 MeSH descriptor: [Quality of Life] explode all trees #9 (physical health OR mental health OR health status OR overall health):ti,ab,kw #10 #7 OR #8 OR #9 #11 #3 AND #6 AND #10 #12 (randomized controlled trial OR RCT):pt #13 #11 AND #12 | MeSH + title/abstract/keyword; RCT publication type |
| Web of Science | TS=((Qigong OR "Qi Gong" OR "Ch'i Kung" OR "Chi Kung")) AND TS=((Aged OR Elderly OR "older adult*" OR Aging)) AND TS=((Health OR "Health Status" OR "Quality of Life" OR "Physical Health" OR "Mental Health")) AND TS=(("Randomized Controlled Trial" OR RCT)) | Topic field; RCT filter |
| CNKI (China National Knowledge Infrastructure) | (SU%=健身气功 OR SU%=气功 OR SU%=八段锦 OR SU%=五禽戏 OR SU%=六字诀 OR SU%=易筋经) AND (SU%=老年人 OR SU%=老人 OR SU%=老年人群 OR SU%=高龄) AND (SU%=健康状况 OR SU%=健康 OR SU%=身体健康 OR SU%=心理健康 OR SU%=生活质量 OR SU%=生命质量) AND (FT%=随机对照试验 OR FT%=随机对照 OR FT%=RCT) | Subject (SU) + full text (FT); RCT only; from inception to May 2025 |
| Wanfang Data | 主题:((健身气功 OR 气功 OR 八段锦 OR 五禽戏 OR 六字诀 OR 易筋经)) and 主题:((老年人 OR 老人 OR 老年人群)) and 主题:((健康状况 OR 健康 OR 身体健康 OR 心理健康 OR 生活质量)) and 主题:((随机对照试验 OR 随机对照 OR RCT)) | Topic field; RCT limit |
| VIP (Chinese Science and Technology Periodical Database) | M=健身气功 OR M=气功 OR M=八段锦 OR M=五禽戏 OR M=六字诀 AND M=老年人 OR M=老人 OR M=老年人群 AND M=健康 OR M=健康状况 OR M=生活质量 OR M=心理健康 AND U=随机对照试验 OR U=随机对照 OR U=RCT | Title/keyword (M) + any field (U); RCT only |

Common notes for all databases:

Time period: from database inception to May 2025

Language: no restriction (English and Chinese studies included)

Participants: older adults (aged ≥60 years or explicitly defined as “elderly” / “老年人”)

Intervention: Health Qigong (including Baduanjin, Wuqinxi, Liuzijue, Yijinjing, etc.)

Study design: randomized controlled trial (RCT) only
